# Distinct Antibody Signatures Associated with Different Malaria Transmission Intensities in Zambia and Zimbabwe

**DOI:** 10.1128/mSphereDirect.00061-19

**Published:** 2019-03-27

**Authors:** Tamaki Kobayashi, Aarti Jain, Li Liang, Joshua M. Obiero, Harry Hamapumbu, Jennifer C. Stevenson, Philip E. Thuma, James Lupiya, Mike Chaponda, Modest Mulenga, Edmore Mamini, Sungano Mharakurwa, Lovemore Gwanzura, Shungu Munyati, Susan Mutambu, Philip Felgner, D. Huw Davies, William J. Moss

**Affiliations:** a Johns Hopkins Malaria Research Institute, Johns Hopkins Bloomberg School of Public Health, Baltimore, Maryland, USA; b Vaccine Research & Development Center, Department of Physiology & Biophysics, School of Medicine, University of California, Irvine, Irvine, California, USA; c Macha Research Trust, Choma, Zambia; d Tropical Diseases Research Centre, Ndola, Zambia; e Biomedical Research and Training Institute, Harare, Zimbabwe; f National Institute of Health Research, Harare, Zimbabwe; University at Buffalo; Ehime University; Institut Pasteur

**Keywords:** *Plasmodium falciparum*, serology, malaria, proteomics, surveillance studies

## Abstract

Antibodies to Plasmodium falciparum are specific biomarkers that can be used to monitor parasite exposure over broader time frames than microscopy, rapid diagnostic tests, or molecular assays. Consequently, seroprevalence surveys can assist with monitoring the impact of malaria control interventions, particularly in the final stages of elimination, when parasite incidence is low. The protein array format to measure antibodies to diverse P. falciparum antigens requires only small sample volumes and is high throughput, permitting the monitoring of malaria transmission on large spatial and temporal scales. We expanded the use of a protein microarray to assess malaria transmission in settings beyond those with a low malaria incidence. Antibody responses in children and adults were profiled, using a P. falciparum protein microarray, through community-based surveys in three areas in Zambia and Zimbabwe at different stages of malaria control and elimination. These three epidemiological settings had distinct serological profiles reflective of their malaria transmission histories. While there was little correlation between transmission intensity and antibody signals (magnitude or breadth) in adults, there was a clear correlation in children younger than 5 years of age. Antibodies in adults appeared to be durable even in the absence of significant recent transmission, whereas antibodies in children provided a more accurate picture of recent levels of transmission intensity. Seroprevalence studies in children could provide a valuable marker of progress toward malaria elimination.

**IMPORTANCE** As malaria approaches elimination in many areas of the world, monitoring the effect of control measures becomes more important but challenging. Low-level infections may go undetected by conventional tests that depend on parasitemia, particularly in immune individuals, who typically show no symptoms of malaria. In contrast, antibodies persist after parasitemia and may provide a more accurate picture of recent exposure. Only a few parasite antigens—mainly vaccine candidates—have been evaluated in seroepidemiological studies. We examined antibody responses to 500 different malaria proteins in blood samples collected through community-based surveillance from areas with low, medium, and high malaria transmission intensities. The breadth of the antibody responses in adults was broad in all three settings and was a poor correlate of recent exposure. In contrast, children represented a better sentinel population for monitoring recent malaria transmission. These data will help inform the use of multiplex serology for malaria surveillance.

## Abstract (precis)

As malaria approaches elimination in many areas of the world, monitoring the effect of control measures becomes more important but challenging. Low-level infections may go undetected by conventional tests that depend on parasitemia, particularly in immune individuals, who typically show no symptoms of malaria. In contrast, antibodies persist after parasitemia and may provide a more accurate picture of recent exposure. Only a few parasite antigens—mainly vaccine candidates—have been evaluated in seroepidemiological studies. We examined antibody responses to 500 different malaria proteins in blood samples collected through community-based surveillance from areas with low, medium, and high malaria transmission intensities. The breadth of the antibody responses in adults was broad in all three settings and was a poor correlate of recent exposure. In contrast, children represented a better sentinel population for monitoring recent malaria transmission. These data will help inform the use of multiplex serology for malaria surveillance.

## INTRODUCTION

Accurate measurement of the burden of malaria and monitoring of the effectiveness of interventions are essential for effective malaria control and elimination programs. Current methods measure the disease incidence or parasite prevalence in children or defined populations using microscopy, rapid diagnostic tests (RDTs), or molecular methods through passive or active case detection (1). Although these methods are useful, they typically provide prevalence estimates over a narrow window of time, unless they are repeated frequently, and may be imprecise in areas with low or changing transmission (2). Serology is an alternative tool for malaria surveillance and provides a measure of parasite exposure over broader time frames than standard diagnostic tests (3), although the patterns, breadth, and duration of parasite antigen-specific antibodies in different transmission settings are not well characterized. Serological surveys have been used to identify spatial heterogeneities in malaria transmission (4–7), monitor the impact of malaria control interventions (8), and characterize malaria transmission intensity (3, 9). Despite its wide application, the choice of antigens for malaria serological surveys has been restricted to vaccine candidates, such as apical membrane antigen 1 (AMA-1), merozoite surface protein 1 (MSP1), or MSP2. These antigens were identified in searches of antigens that induce protective immunity and may not necessarily be good candidates to measure the history of exposure to malaria parasites. The protein microarray is a highly versatile and high-throughput platform to study protein interactions, including, but not limited to, profiling of the serological responses following infection by pathogens of interest. Many studies have used protein microarrays to identify antibody signatures associated with protective immunity to Plasmodium falciparum infection (10–16). However, the application of protein microarray data for disease surveillance is in its infancy (17).

**FIG 1:**
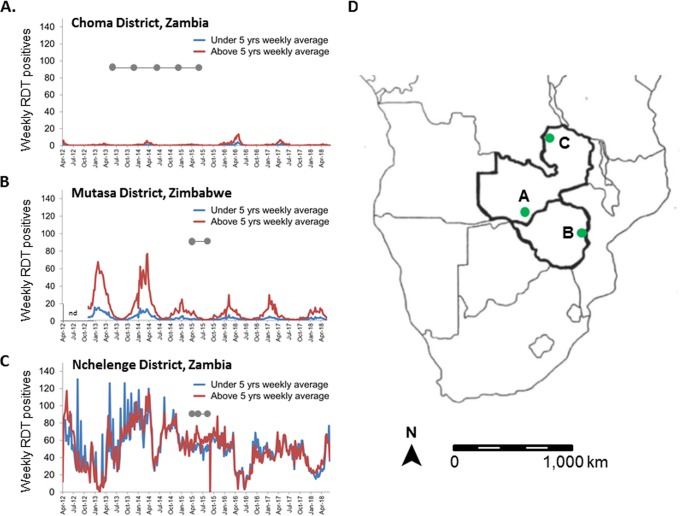
Trends in malaria burden in the three study sites and timing of sample collection for serological studies. (A to C) Charts showing trends in malaria burden over a 6-year period for Choma District, Zambia, with successful malaria control (entomological inoculation rate, <1; prevalence, 1%) (A), Mutasa District, Zimbabwe, with resurgent malaria after previous control, followed by decreasing transmission after indoor residual spraying (entomological inoculation rate, 10; parasite prevalence, 5 to 10%) (B), and Nchelenge District, Zambia, with ineffective malaria control (entomological inoculation rate, 80; parasite prevalence, 50%) (C). The timing of sample collection for the serological assays is indicated by linked dots. (D) A map of South Africa shows the location of the study sites. nd, not done.

To identify the serological profiles associated with different malaria transmission settings, a protein array platform in which expressed proteins are printed onto a support surface was used to measure antibodies to 500 P. falciparum antigens in samples collected in community-based surveys in three transmission settings in Zambia and Zimbabwe: (i) Choma District in Southern Province, Zambia (18), which underwent a dramatic decline in malaria transmission over the past decade and where malaria is nearing elimination; (ii) Nchelenge District in Luapula Province in northern Zambia (18), which has had high, perennial transmission, despite the use of malaria control measures, such as insecticide-treated bed net distribution and focal indoor residual spray (IRS) campaigns; and (iii) Mutasa District in eastern Zimbabwe (18, 19), which borders Mozambique and which experienced a malaria resurgence after successful malaria control, although recent IRS campaigns with pirimiphos-methyl have successfully reduced the malaria burden (20) (Fig. 1). The serological profiles among children and adults in these different ecological and geographical settings reflect the malaria transmission histories and inform progress toward control and elimination.

## RESULTS

### Characteristics of study population.

A total of 429 samples were collected in serial, cross-sectional, community-based surveys from 290 individuals from 3 sites in 2015. Data from 213 individuals were analyzed (Table 1). In the low-transmission setting in Choma District, Zambia, 70 samples from 70 individuals were analyzed. In the intermediate-transmission setting in Mutasa District, Zimbabwe, 156 samples from 78 individuals were analyzed, and in the high-transmission setting in Nchelenge District, Zambia, 124 samples from 69 individuals were analyzed. The age of the participants ranged from younger than 1 year to 86 years, with the median age per site ranging from 15 to 21 years. Fever (tympanic temperature, ≥38°C) on the day of screening was rare among this population across all the transmission intensity levels, indicating that most infected individuals were asymptomatic. As not all residents were home at the time of the study visit, the number of visits per participant varied. Samples collected from 13 adults in Choma District, Zambia (median age at the first visit, 57 years) from 5 time points spanning June 2013 to June 2015 were also analyzed. In addition to the samples collected from community surveys, samples from 35 RDT-positive, symptomatic patients attending rural health centers in the Macha Hospital catchment area in Choma District, Zambia, were analyzed (Table 1).

**TABLE 1:** Characteristics of study sites and participants

Characteristic	Value for the indicated location:				
	Active community surveillance				Clinical samples from Choma District, Zambia
	Serial cross-sectional survey			Choma District, Zambia, complete follow-up for 2 yr	
	Choma District, Zambia	Mutasa District, Zimbabwe	Nchlelenge District, Zambia		
Sampling date(s)	Jun 2015	Apr and Aug 2015	Apr, Jun, and Aug 2015	Jun and Dec 2013, Jun and Dec 2014, Jun 2015	Nov 2013 to May 2015
Malaria transmission	Near elimination	Intermediate	High	Near elimination	Near elimination
No. of participants	70	78	69	13	35
% male participants	51	46	45	54	63
Median (interquartile range) age (yr) at first visit	17 (6–47)	15 (6–33)	21 (9–39)	57 (44–68)	11 (4.4–33)
No. of plasma samples	70	156	124	65	35
% of participants febrile on day of screening	0	0.4	1.7	0	84
% of participants microscopy positive	0	Apr, 1.3; Aug, 1.3	Apr, 27; Jun, 25; Aug, 21	0	NA^*a*^
% of participants RDT positive	0	Apr, 2.6; Aug, 1.3	Apr, 36; Jun, 43; Aug, 27	0	100

a NA, not applicable.

### Overall serological profiles in different malaria transmission settings.

**FIG 2:**
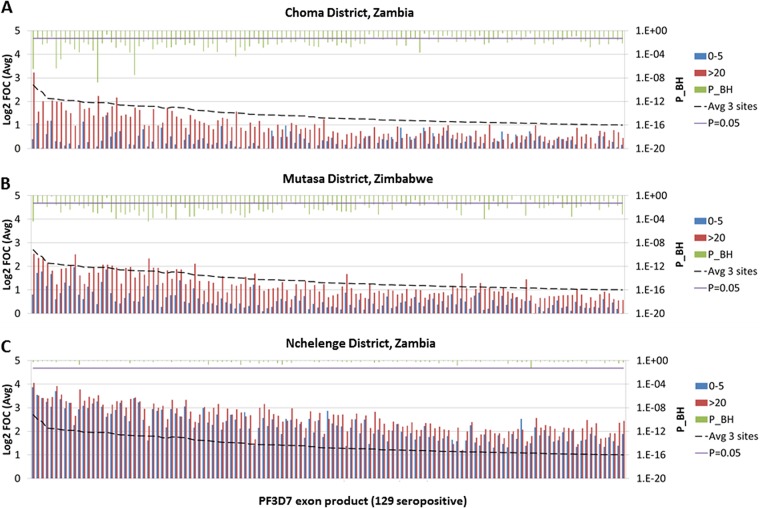
Comparison of Plasmodium falciparum-specific seropositive antigen responses in adults and children. Antibody responses in adults (>20 years old) and children (0 to 5 years old) are arranged from left to right according to the average signal intensity across the three sites. A cutoff log_2_ FOC of 1 was used to define seropositivity. The antigens are listed in the same order in each panel. Of 500 P. falciparum antigens on the array, 129 antigens were seropositive. Benjamini-Hochberg (BH)-corrected *P* values for *t* tests comparing adults and children are shown (secondary *y* axis), and the cutoff value for significance (*P* < 0.05) is indicated.

Of the 500 P. falciparum antigens on the array, the number of antigens recognized in individual participants (breadth of the antibody profile) was highly variable, ranging from less than 10 to more than 300 antigens, depending on the age of the participants and the sites where the samples were collected. Overall, 129 antigens were above the seropositivity threshold (log_2_ fold change in expression over that for the control, i.e., fold over control [FOC], >1) based on the mean signal for the whole study population. Shown in Fig. 2 are the average signal intensities against these 129 seropositive antigens for children (age range, 0 to 5 years) and adults (age, >20 years) in each site. The greatest difference between children and adults was in the low-transmission setting in Choma District (Fig. 2A), whereas the lowest difference was in the high-transmission setting in Nchelenge District (Fig. 2C). Mutasa District showed an intermediate picture (Fig. 2B). Similarly, the number of exon products that were differentially recognized (Benjamini-Hochberg [BH]-corrected *P* value [*P*_BH] < 0.05) by children and adults was 97, 100, and 1 in the Choma District, Mutasa District, and Nchelenge District, respectively (data not shown).

**FIG 3:**
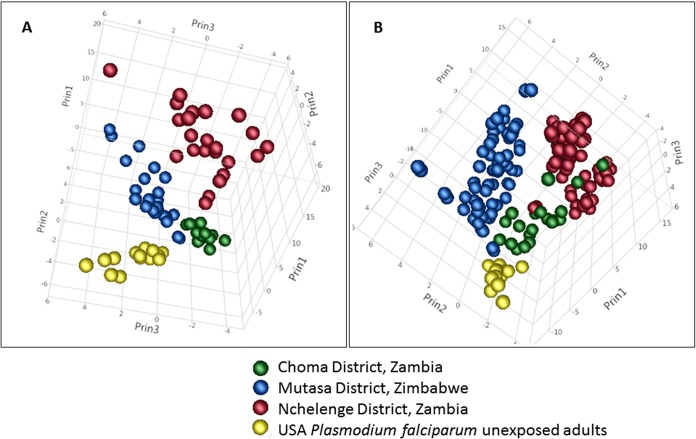
Unsupervised principal-component analysis (PCA) of 129 seroreactive antigens for adults and children from different geographical locations. (A) Children (0 to 5 years old); (B) adults (>20 years old). Samples are color coded in accordance with the geographical location, as indicated on the plot.

Unsupervised principal-component analyses (PCA) of the profiles in children separated the three sites from each other and from U.S. naive controls (Fig. 3A), consistent with the different transmission intensities across the three sites. Despite adults having broader and more overlapping profiles than children, the adult profiles also resolved into distinct clusters according to transmission intensity (Fig. 3B), possibly reflecting a geographical component to the antibody signature. This hypothesis was supported by hierarchical clustering of data for all 500 exon products on the array (see Fig. S1 in the supplemental material). In children, samples from Nchelenge District, Zambia, and Choma District, Zambia, clustered together on the same major limb of the dendrogram, despite having the highest and lowest signals, respectively, while samples from Mutasa District, Zimbabwe, clustered in a separate limb.

10.1128/mSphereDirect.00061-19.1 FIG S1 Hierarchical clustering dendrogram and heat map of protein microarray data for children 0 to 5 years of age from 3 sites of differing malaria transmission intensity. All 500 P. falciparum exon products on the array are represented and ranked from top to bottom by the average signal across the three sample populations. Average linkage hierarchical clustering of serum samples (left to right) was generated with multiexperiment software (http://mev.tm4.org) using Pearson correlations as the distance metric. The results are for Choma District, Zambia (Ch) samples from June 2015 (*n* = 28); Mutasa District, Zimbabwe (Mu), samples from August 2015 (*n* = 15); Nchelenge District, Zambia (Nc), samples from August 2015 (*n* = 19). Download FIG S1, TIF file, 0.5 MB. Copyright © 2019 Kobayashi et al. 2019 Kobayashi et al. This content is distributed under the terms of the Creative Commons Attribution 4.0 International license.

### Magnitude of age-specific serological profiles for the top 30 antigens in different malaria transmission settings.

We then examined the responses to the dominant antigens, defined by ranking all 500 P. falciparum antigens on the array by the average fluorescence signal in descending order. The top 30 dominant antigens (listed in Table S1) included six P. falciparum EMP1 (PfEMP1) exon products mapping to the conserved intracellular domain, five merozoite surface proteins (MSP1, -2, -4, -10, and -11), three early transcribed membrane proteins (ETRAMP2, -5, and -14), and two liver-stage antigens (LSA1 and -2).

10.1128/mSphereDirect.00061-19.7 TABLE S1 Thirty Plasmodium falciparum-reactive exon products with the highest antibody signal intensities. Antigens are ranked by descending average signal intensity across the S. African study population. Shown is a comparison of the top 30 seroreactive P. falciparum antigens ranked using the fold over control (FOC) and two-component mixture model (MM) methods. The asterisk indicates a marker of recent exposure in Uganda, as described by Helb et al. (17). Download Table S1, DOCX file, 0.02 MB. Copyright © 2019 Kobayashi et al. 2019 Kobayashi et al. This content is distributed under the terms of the Creative Commons Attribution 4.0 International license.

**FIG 4:**
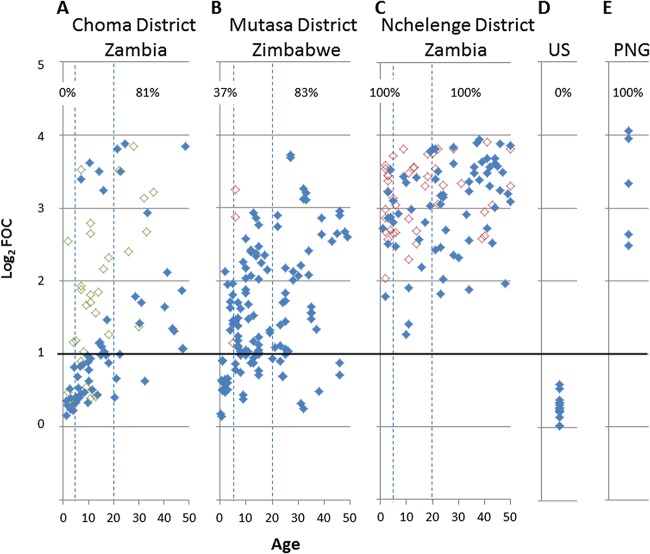
Age-stratified protein microarray data for different transmission settings. (A to C) Scatter plots of the mean signal intensity for the 30 antigens with the highest antibody signal intensities (log_2_ FOC) by age for each transmission setting. Individual symbols represent single serum samples. Samples from RDT-negative and -positive samples are indicated by solid and open symbols, respectively. The RDT-positive samples are colored red and green to distinguish, respectively, active community surveillance samples from samples from patients with malaria presenting to rural health centers in the Macha Hospital catchment area in Choma District. (A) Choma District, Zambia, samples collected from June 2015 (*n* = 70 community surveillance donors, 70 samples, plus *n* = 35 care-seeking individuals, 35 samples). (B) Mutasa District, Zimbabwe, samples collected from April and August 2015 (*n* = 78 donors, 156 samples). (C) Nchelenge District, Zambia, samples collected in April, June, and August 2015 (*n* = 69 donors, 124 samples). Hashed lines signify children (0 to 5 years) and adults (>20 years), with the percentage of RDT-negative samples in each age group above a cutoff of a log_2_ FOC of >1 being shown at the top of each panel. (D) Healthy U.S. adults (*n* = 14). (E) Healthy adults from Papua New Guinea (*n* = 5). The top 30 antigens are listed in Table S1 in the supplemental material.

Scatter plots of the average signal intensity of the top 30 antigens for the 3 settings, as well as signals from adults from the United States (a nontransmission setting) and adults from Papua New Guinea (PNG; a high-transmission setting) (Fig. 4), revealed age- and transmission intensity-dependent patterns similar to those achieved with all 129 seropositive antigens. In the low-transmission setting in Choma District, Zambia, the signals were the lowest in children younger than 5 years of age. None had an average log_2_ FOC above the cutoff of 1, and the signals were similar in magnitude to the corresponding antibody signals in healthy U.S. adults (Fig. 4A and D, respectively), reflecting the low transmission in Choma District. The antibody signal intensity increased with increasing age, with 81% of adults in Choma District older than 20 years of age seropositive for the highest-intensity 30 antigens. These may represent the durable antibody responses generated when transmission was higher in this location.

In the resurgent malaria transmission setting in Mutasa District, Zimbabwe, antibody signals for the highest-intensity 30 antigens also increased with age (Fig. 4B) but were higher than those in the low-transmission setting in Choma District, Zambia, particularly among children and adolescents. Thirty-seven percent of children younger than 5 years of age and 83% of adults older than 20 years of age were seropositive for the highest-intensity 30 antigens.

In the high-transmission setting in Nchelenge District, Zambia, antibody signals were high across all ages, with all participants, including children younger than 5 years of age, being seropositive for the highest-intensity 30 antigens (Fig. 4C). These signals were comparable in magnitude to those of samples from PNG.

### Breadth of antigen-specific antibody responses by age in different malaria transmission settings.

The results of analysis of the antibody responses to the individual top 30 antigens are shown in Fig. S2. These mirrored the average signal data described above and further highlight age-specific differences in the breadth of the antibody responses in different transmission settings. In the low-transmission setting in Choma District, Zambia, children younger than 5 years of age not only had the lowest antibody signal intensities but also had the narrowest antibody profiles, being seropositive for only 5 of 500 P. falciparum antigens (Fig. S2). The antibody breadth increased 5-fold in children 6 to 10 years of age (seropositive to 25 antigens) and 15-fold in individuals 11 to 20 years of age. Adults between 20 and 40 years of age were seropositive to 153 of 500 antigens. In the intermediate-malaria-transmission setting in Mutasa District, Zimbabwe, the antigen-specific antibody signal intensity similarly increased with age, but the breadth of the antibody response was greater in each age group in this setting than in the low-transmission setting. Specifically, children younger than 5 years of age were seropositive to 15 antigens and children 6 to 10 years of age were seropositive to 53 antigens. In contrast to the low- and intermediate-transmission sites, the acquisition of antigen-specific antibodies in Nchelenge District, Zambia, was rapid. Children younger than 5 years of age had antigen-specific antibody profiles that were approximately 80% of those of adults.

10.1128/mSphereDirect.00061-19.2 FIG S2 Age-associated changes in antibody profiles. Age-stratified signal intensities for the 30 antigens with the highest antibody signal intensities for the three transmission settings, as well as U.S. healthy controls (US; adults) and individuals from an additional high-transmission setting in Papua New Guinea (PNG; adults). Individual antigens are represented by different symbols. The number of seropositive antigens out of 500 on the P. falciparum array above a cutoff of a log_2_ FOC of >1 are shown at the top of each plot. Download FIG S2, TIF file, 0.2 MB. Copyright © 2019 Kobayashi et al. 2019 Kobayashi et al. This content is distributed under the terms of the Creative Commons Attribution 4.0 International license.

### Magnitude of antibody signals associated with parasitemia.

It was of interest to determine the effect of infection with P. falciparum on the magnitude of antibody signals and the breadth of antibody profiles. Therefore, we compared RDT-positive and -negative samples for the 3 sites (open and filled symbols in Fig. 4, respectively). For Choma District, since none of the community surveillance participants were RDT positive, Fig. 4 shows the signals for 35 RDT-positive malaria cases (age range, 2 to 78 years; median age, 11 years) attending health care facilities in the catchment area of Macha Hospital, Choma District. In Mutasa District, 0.9 to 4.1% and 27% to 43% of Nchelenge District participants were RDT positive, depending on the time of sample collection (Table 1). Overall, RDT-positive individuals had elevated antibody signals relative to age-matched RDT-negative individuals in all three sites, consistent with the findings of similar studies performed elsewhere (21–24). The differential was the greatest in individuals in Choma District, particularly in children, where the background seroreactivity was the lowest. Thus, for children aged 0 to 10 years, parasitemia was associated with elevated antibody signals on the array, with a mean log_2_ FOC of 0.41 for RDT-negative individuals (*n* = 30) and 1.44 for RDT-positive individuals (*n* = 12). In this age group there was also an increase in the breadth of the antibody profiles associated with parasitemia, ranging from 9 to 57 seropositive antigens (Fig. S3). Of the 57 seropositive antigens recognized by RDT-positive participants, 19 were differentially recognized (*P*_BH < 0.05) between those who were RDT positive and those who were RDT negative (listed in Table 2) and could be potential serodiagnostic antigens. In Nchelenge District, the differential between the RDT-positive and -negative participants was marginal (mean log_2_ FOC = 3.1 and 3.2, respectively) owing to the elevated signals found in RDT-negative individuals.

**TABLE 2:** Antigens differentially recognized by RDT-negative and RDT-positive participants from Choma District, Zambia^*a*^

PF3D7 gene identifier	Exon	Name	SI for the following participants:		*P_*BH
			RDT negative (*n* = 30)	RDT positive (*n* = 12)	
0532100		ETRAMP5	0.86	2.17	0.0169
0800200	Exon 2, segment 1	PfEMP1	0.39	2.07	0.0169
0220000	Exon 2, segment 2	LSA3	0.58	1.98	0.0140
1007700	Exon 1, segment 2	ApiAP2	0.47	1.95	0.0428
0223300		PfEMP1	0.47	1.88	0.0169
1300300	Segment 2	PfEMP1	0.52	1.85	0.0140
0315400	Exon 1 of 1	Unknown function	0.60	1.79	0.0169
0903500	Exon 1, segment 1	Unknown function	0.37	1.71	0.0140
1222600		ApiAP2	0.54	1.61	0.0169
1033200	Exon 1, segment 1	ETRAMP10.2	0.35	1.58	0.0140
1121800	Segment 1	Peptidase, M16 family	0.64	1.43	0.0241
1301400	Exon 2 of 2	Hyp12, unknown function	0.49	1.36	0.0367
0817300	Exon 1, segment 3	Asparagine-rich antigen	0.38	1.35	0.0169
1353100	Exon 2, segment 1	Exported protein, unknown function	0.10	1.27	0.0140
0711700	Exon 2, segment 1	PfEMP1	-0.23	1.27	0.0169
0808600	Exon 2, segment 1	PfEMP1	0.17	1.20	0.0394
0301700	Exon 2, segment 1	Exported protein, unknown function	0.29	1.18	0.0169
1109200	Exon 3 of 4	Conserved protein, unknown function	0.38	1.15	0.0367
0202000	Exon 2, segment 1	KAHRP	0.24	1.07	0.0140

a SI, signal intensity (log_2_ FOC); RDT, rapid diagnostic test; *P*_BH, Benjamini-Hochberg [BH]-corrected *P* value (*P* value corrected for false discovery); ETRAMP, early transcribed membrane protein; LSA, liver-stage antigen; PfEMP, P. falciparum erythrocyte membrane protein; ApiAP2, transcription factor with an AP2 domain(s); KAHRP, knob-associated histidine-rich protein. Data are shown in Fig. S3 in the supplemental material

10.1128/mSphereDirect.00061-19.3 FIG S3 Comparison of Plasmodium falciparum-specific seropositive antigen responses in RDT-negative and RDT-positive children (age, 0 to 10 years) in Choma District, Zambia. Antibody responses in RDT-negative (blue bars, *n* = 30) and RDT-positive (red bars, *n* = 12) individuals aged 10 years or younger, arranged from left to right according to average signal intensity. *P* values with Benjamini-Hochberg correction for false discovery (*P*_BH) for *t* tests comparing RDT-positive and -negative individuals are shown in green (secondary *y* axis), and the cutoff value for significance (*P* < 0.05) is indicated by a hashed purple line. Antigens are segregated into those that are differentially recognized (*P*_BH < 0.05), shown on the left (*n* = 19), and nondifferentially recognized (*P*_BH ≥ 0.05), shown on the right. Antigens are considered seropositive if the log_2_ FOC is ≥1. The 19 differentially reactive/seropositive antigens are listed in Table 2. Download FIG S3, TIF file, 0.1 MB. Copyright © 2019 Kobayashi et al. 2019 Kobayashi et al. This content is distributed under the terms of the Creative Commons Attribution 4.0 International license.

### Children younger than 5 years of age as a sentinel surveillance population.

**FIG 5:**
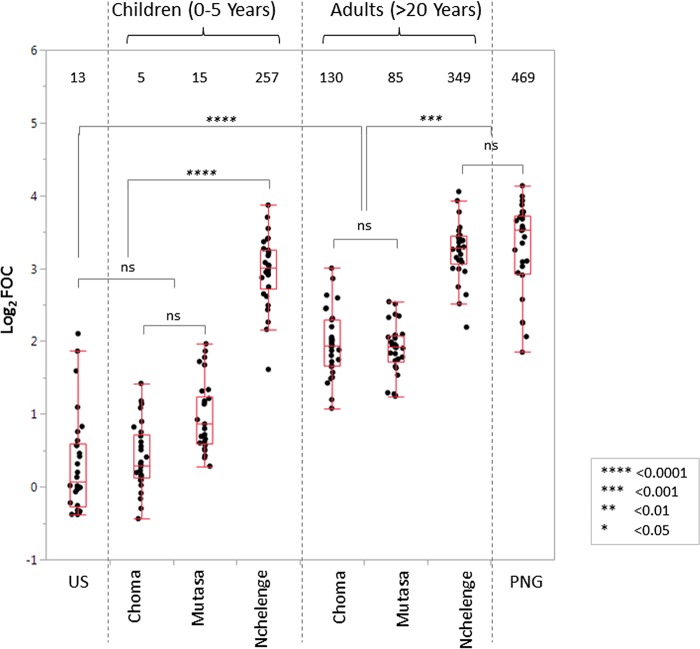
Comparisons of antibody signal intensities for the top 30 antigens between children and adults. Box plots of antibody signal intensities for the top 30 antigens that gave the highest antibody signal intensities across the three transmission settings, as well as for U.S. healthy controls (US; adults) and individuals from a high-transmission setting in Papua New Guinea (PNG; adults). *P* values for nonparametric comparisons were adjusted using Dunn’s correction. All possible pairwise comparisons are listed in Table S2 in the supplemental material. The number of seropositive antigens out of 500 on the P. falciparum array above a cutoff of log_2_ FOC >1 is shown at the top of each plot.

The utility of children younger than 5 years of age as a sentinel surveillance population for serological monitoring of ongoing malaria transmission and documentation of elimination was tested by comparing the signals to the top 30 antigens in children and adults across the three transmission settings and in the U.S. and PNG controls (Fig. 5; Table S2). The signals from children in Choma District and U.S. adults were not significantly different, consistent with the low transmission in Choma District. Although children in Mutasa District had a higher median signal to the top 30 antigens than children in Choma District, consistent with reemergence in Mutasa District prior to the recent decline, the difference was not significant.

10.1128/mSphereDirect.00061-19.8 TABLE S2 *P* values for all pairwise comparisons shown in Fig. 5 between adult and children IgG profiles. Download Table S2, DOCX file, 0.2 MB. Copyright © 2019 Kobayashi et al. 2019 Kobayashi et al. This content is distributed under the terms of the Creative Commons Attribution 4.0 International license.

The signals from children from Choma and Mutasa Districts were highly significantly different from those from children in Nchelenge District, consistent with the higher transmission in the latter setting. Unlike the signals from children, signals from adults were less discriminatory, with the distribution of the average signals for the top 30 antigens being indistinguishable between Choma and Mutasa Districts. The signals from adults in both districts were significantly lower than those from adults in Nchelenge District. Antigen-specific antibody signal intensities among adults in the high-transmission setting in Nchelenge District, Zambia, were not significantly different from those in a high-transmission reference sample from PNG. Antigen-specific antibody signal intensities among adults in the low-transmission setting in Choma District, Zambia, and the intermediate-transmission setting in Mutasa District, Zimbabwe, were lower than those in the high-transmission setting but higher than those in U.S. adults.

With the aim of achieving improved discrimination between children in low- and intermediate-transmission settings (Choma District and Mutasa District, respectively), signals for individual top 30 antigens were compared using both *t* tests and receiver operating characteristic (ROC) analyses (Fig. S4). A number of discriminatory antigens produced significantly higher signals in children from Mutasa District than in children from Choma District. Four of these yielded area-under-the-ROC-curve (AUC) values of >0.9; specifically, these were ApiAP2 (0.976), PfEMP1 (0.963), SERA4 (0.919), and a protein of unknown identity (PF3D7_0903500). Further experiments using purified protein antigens in other immunoassay formats, such as enzyme immunoassays, would be needed to validate these as potential serodiagnostic antigens for recent exposure.

10.1128/mSphereDirect.00061-19.4 FIG S4 ROC analysis and *t* test comparisons of top 30 antigens identify antigens associated with different transmission intensities. Shown are AUC and *P* values for 3 different site comparisons (Nchelenge District versus Choma District, Nchelenge District versus Mutasa District, and Mutasa District versus Choma District). In each ROC comparison, the analysis was performed twice, with the status for each site defined as 0 or 1, although only AUC values of >0.5 are shown. For Mutasa District versus Choma District, the AUC values apply to a Mutasa District status of 1, while those marked with an asterisk are applies to a Choma District status of 1. Antigens with an AUC of >0.8 in a column for a site with a status of 1 were considered discriminatory for that site. *P* values with the Benjamini-Hochberg correction for false discovery (*P*_BH) that are <0.05 are colored red. Download FIG S4, TIF file, 0.1 MB. Copyright © 2019 Kobayashi et al. 2019 Kobayashi et al. This content is distributed under the terms of the Creative Commons Attribution 4.0 International license.

### Antibodies persist after reduction of malaria transmission.

**FIG 6:**
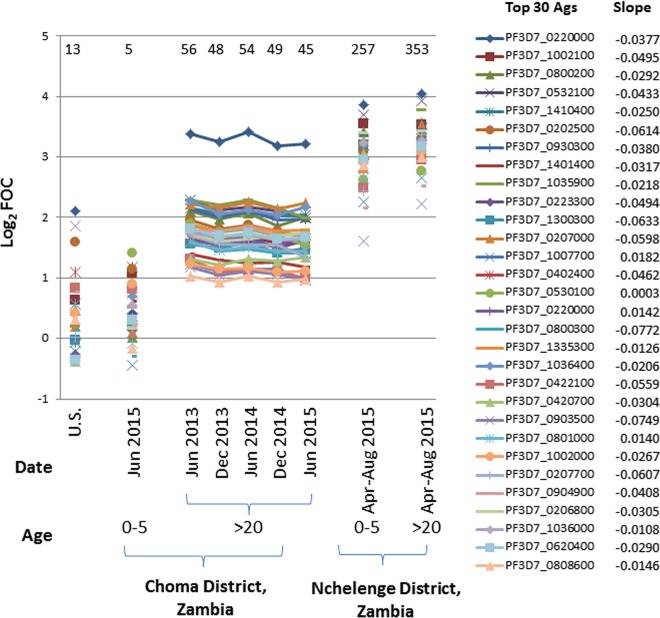
Persistent antibody responses among adults in Choma District, Zambia, to the 30 antigens (Ags) with the highest antibody signals. Mean antibody signal intensities for the 30 antigens with the highest antibody signal intensities are shown for five time points over 2 years in 13 adults (age range, 35 to 82 years; median age, 60 years) residing in the low-transmission setting in Choma District, Zambia. Slope refers to the slope of the regression line through the 5 time points for each antigen. For comparison, mean antibody signal intensities for the same top 30 antigens are shown for children (age, 0 to 5 years) and adults (age, >20 years) residing in the high-transmission setting of Nchelenge District, Zambia. Individual antigens are represented by different symbols. The number of seropositive antigens out of 500 on the P. falciparum array above a cutoff of a log_2_ FOC of >1 is shown at the top of each plot.

The prevalence of antibodies to P. falciparum in adults in Choma District, Zambia, following a dramatic decline in malaria transmission over the past decade suggests long-term antibody persistence. This was confirmed by measuring antibody responses to the 30 antigens with the highest signal intensity in a cohort of 13 adults from Choma District, Zambia, from whom samples were assayed at five time points between June 2013 and June 2015 (Fig. 6). The magnitude of the responses to the 30 antigens with the highest signal intensity was stable during the 2-year period, as was the breadth of the antibody profile. However, slopes for 26 of the 30 antigens were negative (data not shown), consistent with some waning of antibody levels.

### Validation of diagnostic antigens.

Analysis of the data using the two-component mixture model generated a list of 30 dominant antigens (Table S1), of which 27 were also on the top 30 antigen list using the FOC method. This provides a useful cross-validation using two independent analytical methods. One of the goals of this study was to identify and validate potentially useful biomarkers for monitoring recent exposure to P. falciparum. Several reactive antigens identified among corresponding *in vitro* transcription/translation (IVTT)-expressed exon products were also represented by Escherichia coli-derived purified proteins on the same array (Table S3), namely, EBA175, MSP1, MSP2, LSA1, and CSP. Scatter plots of array signal intensity data for purified protein versus an IVTT-expressed exon product(s) were generated (Fig. S5). Good correlations were seen for MSP2 (*R*^2^ = 0.89) and EBA175 (*R*^2^ = 0.75), and modest correlations were seen for CSP (*R*^2^ = 0.6), LSA1 (*R*^2^ = 0.57), and MSP1 (*R*^2^ = 0.58 for IVTT segment 2 and *R*^2^ = 0.47 for IVTT segment 1). The highest correlation was seen for MSP2, where both the IVTT and purified versions were full-length proteins. Full-length PfCSP was also represented as an IVTT protein and a purified protein, although the reactivity to the purified protein was considerably higher than that to the IVTT protein, with a correspondingly lower correlation. This may reflect the low expression levels of this particular protein in the IVTT system. EBA175 also gave relatively good correlations, despite the different lengths of proteins used. Thus, for IVTT expression, exon 1 (4,263 bp) was amplified and expressed as two half-size segments (segments S1 and S2) and printed separately. Shown in Fig. S5 is the correlation of the antibody signals obtained using IVTT-expressed S2 (corresponding to residues 702 to 1421) and purified EBA175 conserved regions RIII to RV (25, 26), corresponding to residues 761 to 1298 (27). The IVTT expression product overlapped the entire RIII to RV purified protein fragment, and any discrepancies in the correlation may be accounted for by the additional 59 and 123 amino acids at the N- and C-terminal ends of the IVTT expression product, respectively, that may provide epitopes for antibody recognition that are absent from the RIII to RV fragment. MSP1 gave weak correlations. For IVTT expression, the single exon of MSP1 (5,163 bp) was amplified in two segments (segments S1 and S2) corresponding to the N- and C-terminal 870 and 868 residues, respectively, and printed separately. Figure S5 shows the correlation obtained using IVTT-expressed segment S1 or S2 compared to purified MSP1-19 (corresponding to residues 1607 to 1702 of MSP1) glutathione *S*-transferase fusion protein. Segment S1 and MSP1-19 do not overlap, which presumably accounts for the weakest correlation seen in Fig. S5. Although segment S2 completely overlaps MSP1-19, the IVTT exon product has an additional ∼770 residues that may provide epitopes for antibody recognition that are absent from the MSP1-19 fragment. Finally, for IVTT expression of LSA1, the single exon (3,489 bp, 1,162 amino acids) was amplified in two segments. Segment 1 of 2 contains the N-terminal 250 residues. Figure S5 shows the correlations of the antibody signals obtained using IVTT segment 1 and the purified LSA-NRC vaccine antigen, which comprises the N- and C-terminal regions of LSA1 (residues 28 to 154 and 1630 to 1909) with two 17-amino-acid repeats located between (28). The correlation between IVTT protein and purified protein was weak, presumably because the IVTT expression product lacks the C-terminal region that may provide additional epitopes for antibody recognition that are absent from the IVTT-expressed segment. Given that many of the IVTT-expressed versions were exon products and not full-length proteins, strong correlations are not expected for all antigens.

10.1128/mSphereDirect.00061-19.5 FIG S5 Scatter plots for protein microarray data for the IVTT-expressed exon product and the corresponding purified protein. Download FIG S5, TIF file, 0.1 MB. Copyright © 2019 Kobayashi et al. 2019 Kobayashi et al. This content is distributed under the terms of the Creative Commons Attribution 4.0 International license.

10.1128/mSphereDirect.00061-19.9 TABLE S3 Comparison between purified proteins and corresponding IVTT-expressed exon products. Correlations are shown in Fig. S5. Download Table S3, DOCX file, 0.02 MB. Copyright © 2019 Kobayashi et al. 2019 Kobayashi et al. This content is distributed under the terms of the Creative Commons Attribution 4.0 International license.

Additional validation using purified proteins, preferably of equivalent length, is needed. Nevertheless, these arrayed purified proteins also reflected the same age- and transmission intensity-dependent patterns seen with the average top 30 IVTT expression exon products (Fig. S6). Thus, all five antigens revealed significantly higher signals in children from Nchelenge District than in children from Choma District and Mutasa District, while signals in adults in Nchelenge District were not significantly higher than those in adults at the other two sites.

10.1128/mSphereDirect.00061-19.6 FIG S6 Dot plots of array data from five purified protein antigens with serum samples from sites with three different transmission intensities. (A) Children ages 0 to 5 years; (B) adults ages >20 years. Download FIG S6, TIF file, 0.2 MB. Copyright © 2019 Kobayashi et al. 2019 Kobayashi et al. This content is distributed under the terms of the Creative Commons Attribution 4.0 International license.

## DISCUSSION

A protein array platform was used to characterize age-specific serological profiles and the breadth of antibody responses to 500 P. falciparum antigens in three transmission settings in Zambia and Zimbabwe. Based on the 30 antigens with the highest signal intensity, distinct age-specific patterns that reflected recent and past P. falciparum transmission levels were identified. Thus, young children in the low-transmission setting of Choma District, Zambia, had antibodies to the fewest P. falciparum antigens. This reflected the recent decline in malaria transmission and the sustained low malaria transmission and could lead to methods to document progress toward elimination. By comparison, adults in the low-transmission setting had high antibody signals, which persisted for the 2 years of a longitudinal study, highlighting their durability. We speculate that these are a legacy of the hyperendemic malaria that existed in this area over a decade ago but could reflect periodic exposure to P. falciparum in the absence of clinical disease. Asymptomatic and low-parasitemic infection occurs even in the setting of declining malaria transmission in Choma District, Zambia. Community-based epidemiological surveillance studies reported a low but persistent parasite prevalence in the population (29, 30). Both studies reported that approximately half of individuals with parasitemia by PCR had insufficient parasite levels to be detected by RDTs (subpatent infection) and that most were asymptomatic. Persistent antibody levels in adults from Choma District may therefore reflect more recent subpatent infection. Interestingly, adults in the resurgent setting of Mutasa District, Zimbabwe, had lower antibody signals and breadth than similarly aged individuals in Choma District, Zambia, again, likely reflecting higher levels of malaria transmission in Choma District, Zambia, more than a decade ago. In contrast, in the high-transmission setting of Nchelenge District of Zambia, the antibody signal intensities for children younger than 5 years of age resembled those for adults from the same setting, reflecting the persistent high transmission.

Numerous studies have characterized serological responses to P. falciparum antigens in sub-Saharan Africa, but none have measured antibodies to 500 P. falciparum proteins across such divergent transmission settings using the same protein array platform. Recently, serological studies measuring antibody responses to malaria vaccine candidates, such as apical membrane antigen 1 (AMA-1) and merozoite surface protein 1 (MSP1) blood-stage antigen, were used to identify the spatial heterogeneity in malaria transmission that could be used to target malaria interventions (31). In a preelimination setting in Swaziland, where only 2 individuals among 4,330 participants were found to be parasitemic by PCR, pooled PCR and serology were shown to be valuable surveillance tools (32). Seropositivity to AMA-1 and MSP1_42_ were associated with age older than 20 years and recent travel to Mozambique (32). Across different transmission settings in Uganda, serological responses to AMA-1 were a better metric of transmission intensity than anemia or parasitemia (33).

The full breadth of reactive antigens in malaria was not fully appreciated until proteome-wide screening techniques, such as the multiplex microarray, were introduced (34–36). The array revealed many novel antigens with potential diagnostic and vaccine applications (11, 14, 21, 37, 38). The array format is also high throughput and inherently sample sparing, yet the real strength in the multiplex format lies in the collective profile of the reactive antigens that it generates, which frequently allows for discrimination between patient groups, such as children with and without clinical immunity (11, 15), or recent and past exposure (17), superior to that which can be achieved from individual antigens alone. Measurement of antibodies to 500 P. falciparum proteins across divergent transmission settings using the same protein array platform provides a collective profile of reactive antigens. The dominance of antibodies to blood-stage antigens is consistent with the findings of other antibody profiling studies of naturally acquired immunity to P. falciparum in Africa (22, 23).

Age-specific seroprevalence profiles can be a useful marker of declining malaria transmission, as was shown in the Gambia and Guinea-Bissau (39), and seroprevalence in young children serves as the best indicator of recent malaria transmission. In our low-transmission setting in Choma District, Zambia, where malaria transmission has declined over the past decade, none of the study participants younger than 5 years of age were seropositive, whereas 81% of adults were seropositive, reflecting the recent changes in malaria transmission. Other studies in sub-Saharan Africa have similarly demonstrated the value of serosurveillance in young children. For example, declining malaria transmission in the Gambia was captured by detection of a decline in seroprevalence in children 1 to 5 years of age (40).

One limitation of this analysis is the focus on antibody responses to the 30 antigens with the highest signal intensity, perhaps selecting for antibodies with longer half-lives and obscuring important but less intense antibody responses with more variable decay kinetics. Among adults residing in the low-transmission setting in Choma District, Zambia, antibodies to the 30 antigens with the highest signal intensity persisted long after the decline in malaria transmission over the past decade. In contrast, of the 14 P. falciparum antigens with short-lived antibody responses identified as markers of recent exposure in Ugandan children (17), 6 were represented among the 30 antigens with the highest signal intensity. The identification of antibodies to P. falciparum antigens with rapid decay kinetics may assist in the detection of recent exposure in preelimination settings. We previously measured serological responses to whole-parasite lysate in the low-transmission setting in Choma District, Zambia, and described increasing seroprevalence with age as well as a spatial overlap between areas of seropositivity and malaria risk based on parasite prevalence (41).

Multiplex serology is a more sensitive measure of exposure than other methods that depend on the presence of parasites (22, 23). There is an extensive literature on the use of serology for surveillance in malaria (42, 43), and wider use of the protein microarray technology to leverage these advantages will require a solution more deployable than conventional confocal laser scanners. To this end, we recently validated a portable digital complementary metal oxide semiconductor (CMOS) array imaging system as a low-cost and robust alternative that could easily be deployed in laboratory settings where the purchase and maintenance of a more complex machine would be cost prohibitive or impractical (44). We also reported that the probing protocol was readily translated to other labs, as shown by excellent correlations between the data generated by five independent laboratories (45). With the availability of training and commercial sources of arrays, the microarray technology can be disseminated more widely, particularly in areas of endemicity, where it will have the greatest impact on public health.

In summary, we showed that protein arrays permit measurement of antibody responses to hundreds of parasite antigens to identify antigen-specific antibody responses in different transmission settings and with different decay kinetics. Using samples obtained from divergent epidemiological settings, the 500-P. falciparum-protein microarray used in this study captured distinct serological profiles reflective of their malaria transmission histories. Furthermore, antibodies in adults persisted in the absence of significant recent transmission, but antibodies in children provided an accurate picture of recent changes in transmission intensity. Seroprevalence studies in children could be used to target malaria interventions and provide a valuable marker of progress toward malaria elimination.

## MATERIALS AND METHODS

### Study sites.

Figure 1 shows the locations and trends in malaria transmission over time in each of the three study sites. Weekly rapid diagnostic test (RDT)-positive counts from local health facilities in individuals younger than 5 years of age and over 5 years of age were reported to the Southern Africa International Centers of Excellence in Malaria Research (ICEMR) and plotted (46–48). The low-malaria-transmission site (Fig. 1A) consisted of the catchment area of the Macha Mission Hospital, a rural settlement in Choma District, Southern Province, Zambia. The area has a tropical savannah climate with cool (May to July) and hot (August to October) dry seasons and a single rainy season (November to April). Malaria transmission is highest during the rainy season, with Anopheles arabiensis mosquitoes being the primary malaria vector (19, 49). Malaria transmission in Southern Province, Zambia, has declined over the past 15 years (19), with a parasite prevalence of approximately 1% by RDT.

The intermediate-malaria-transmission site (Fig. 1B) was Mutasa District, Manicaland Province, Zimbabwe, which lies in the mountainous area of eastern Zimbabwe bordering Mozambique. The seasonal rains are similar to those in Zambia, and the primary malaria vector is A. funestus mosquito (50). Mutasa District had an effective malaria control program from the early 1950s to early 1990s (47), but due to financial constraints, the malaria control program was halted and a resurgence of malaria occurred. Recent annual IRS campaigns using pirimiphos-methyl that began in 2014 successfully reduced the burden of malaria (20).

The high-malaria-transmission site (Fig. 1C) was in Nchelenge District, Luapula Province, Zambia, which shares a border in the west with the Democratic Republic of the Congo and lies in the marshlands of the Luapula River and Lake Mweru. The pattern of rain follows that of Southern Province, Zambia, although the monthly precipitation in Nchelenge District is higher. A. funestus and A. gambiae mosquitoes are the major malaria vectors, and the extended marshlands provide mosquito breeding sites throughout the year (51), resulting in perennial, holoendemic transmission. Despite intensified malaria control efforts, including IRS campaigns with pirimiphos-methyl, malaria transmission remains high (48), with parasite prevalence, as determined by RDT, being higher than 50% throughout the year.

### Sample and data collection.

Simple and stratified random sampling was used to select households for participation in community-based, cross-sectional, and longitudinal surveys as previously described (2). Briefly, households were randomly selected using high-resolution satellite images of the study area. All household residents present at the time of the visit were eligible for enrollment, and written informed consent was obtained from those who agreed to participate and from the caregivers of children. Household visits for the serological studies took place every 6 months between June 2013 and December 2015 in Choma District, Zambia, every 2 months between February and August 2015 in Nchelenge District, Zambia, and every 4 months between February and August 2015 in Mutasa District, Zimbabwe.

To screen for malaria at the household, a PfHRP2-based RDT (SD Bioline Malaria Ag P.f; Standard Diagnostics Inc., Republic of Korea) was performed using blood samples collected by finger prick. Artemether-lumefantrine was offered to treat RDT-positive male and nonpregnant female participants. For serological testing, blood was collected by finger prick using a capillary tube (Microvette CB300; Sarstedt, Nümbrecht, Germany) and stored on ice packs until it was transported to a local laboratory. The blood sample was centrifuged at 14,000 rpm for 1 min to separate the plasma. Plasma samples were stored at −80°C until they were shipped to the University of California, Irvine, on dry ice, where they were again stored at −80°C until probing.

### Protein microarrays.

A custom protein microarray was used to measure antibody responses to P. falciparum as described previously (34). Briefly, 500 exons from P. falciparum (strain 3D7) were amplified from genomic DNA and cloned into a T7 expression vector using homologous recombination. Each was expressed in individual E. coli-derived *in vitro* transcription/translation (IVTT) reactions (5-Prime GmbH, Hilden, Germany) and printed directly onto nitrocellulose-coated glass slides (Grace Bio-Labs Inc., Bend, OR). In addition to the 500 exon products, each array also contained 24 IVTT control (IVTTc) reactions that lacked expression plasmids. Also printed on the array were several purified proteins designed to provide a useful bridge between the array platform and other assay formats that use purified antigens. These antigens included recombinant PfCSP (a gift of Ashley Birkett, PATH) (52, 53), PfLSA1 deleted of the internal repeat domain (a gift of David Lanar, WRAIR) (28), and PfEBA175, PfEBA140, PfMSP1, PfMSP2, PfMSP3, PfRh2, and two long synthetic peptides of PfRh1 (all gifts of James Beeson, Burnet Institute, Melbourne, Australia) (25). Each IVTT expression product contained N-terminal polyhistidine and C-terminal hemagglutinin epitope tags to monitor expression for quality control.

Array probing and data acquisition were performed as described previously (54). To minimize interexperimental variability and array print-batch effects, data were obtained by probing the samples at the same time on the same batch of arrays. Also probed were samples from 15 healthy U.S. adult controls and highly reactive RDT-negative sera from semi-immune adults from Papua New Guinea (PNG).

Raw data were collected as the median pixel signal intensity for each spot subtracted automatically from the local background. Data were normalized using the formula log_2_[IVTT − (SM − GM)]/GM, where IVTT is the exon product signal, SM is the median of the sample-specific IVTTc signals, and GM is the global median of the IVTTc signals. Fold over control (FOC) normalization provides relative measures of the specific antibody binding to the nonspecific antibody binding to the IVTT controls. Thus, with the normalized data, a value of 0.0 means that the intensity is not different from the background intensity and a value of 1.0 indicates a doubling with respect to the background. A threshold of a log_2_ FOC of 1 was used to define seropositivity.

To compare the antibody responses between groups, two metrics were used: (i) magnitude, defined as the log_2_ FOC for each antigen, in which antigens were ranked by the mean signal across the whole study population and the mean for the top 30 was used for comparisons between different age groups and settings, and (ii) the breadth of the antibody profile, defined as the number of seropositive antigens (log_2_ FOC > 1) recognized by an individual or for a population using the mean signal intensity for the population. Differentially reactive proteins between groups were determined using signal intensities and a Bayes regularized *t* test for protein arrays (55). To account for multiple-comparison conditions, the Benjamini and Hochberg (BH) method was used to control the false discovery rate to give corrected *P* values (*P*_BH) (56); a *P*_BH of <0.05 was considered significant. Receiver operating characteristic (ROC) analyses and the area under the ROC curve (AUC) were determined using the R statistical environment and the ROCR tool. Graphical outputs were produced in JMP (SAS Institute, Inc., Cary, NC) and Excel (Microsoft, Redmond, WA) software. Principal-component analyses (PCA) on seroreactive antigens were conducted using JMP software to visually summarize signal intensity variance among the subject groups.

In parallel with the FOC method described above, a two-component mixture model was also used to rank antigens. Reactivity against all 500 exon products at all time points for each subject was applied to the mixture model using an R package (mixtools) for the R statistical environment (57). A subject-specific reactivity cutoff point was defined by the mean +3 standard deviations for the low-reacting subpopulation distribution. An exon product with a reactivity above the cutoff point was identified as positive, and the total number of positive responses per participant defined the breadth of the profile for that subject. Antigens that induced a positive response in more than 25% of the total number of participants were defined as reactive at the population level. Reactive antigens were then ranked on the average signal intensity.

### Ethical considerations.

The study was approved by the Institutional Review Board at the Johns Hopkins Bloomberg School of Public Health, the Tropical Diseases Research Center Ethics Review Committee in Ndola, Zambia, for studies in Choma District and Nchelenge District, Zambia, and the Biomedical Research and Training Institute Institutional Review Board and the Medical Research Council of Zimbabwe for studies in Mutasa District, Zimbabwe. Participants provided written informed consent for themselves and their children.

### Accession number(s).

The full content of the array (which is designated PfPv500) was deposited with the Gene Expression Omnibus database (www.ncbi.nlm.nih.gov/geo/) under accession number GPL18316, and the array data obtained in this study are publicly available at the PlasmoDB Plasmodium Genomics Resource (plasmodb.org).
