# Supplementary figures and images for: Distinct Antibody Signatures Associated with Different Malaria Transmission Intensities in Zambia and Zimbabwe
Source: mSphere. 2019 Mar 27;4(2):e00061-19. doi: 10.1128/mSphereDirect.00061-19 (PMC6437277; doi:10.1128/mSphereDirect.00061-19)

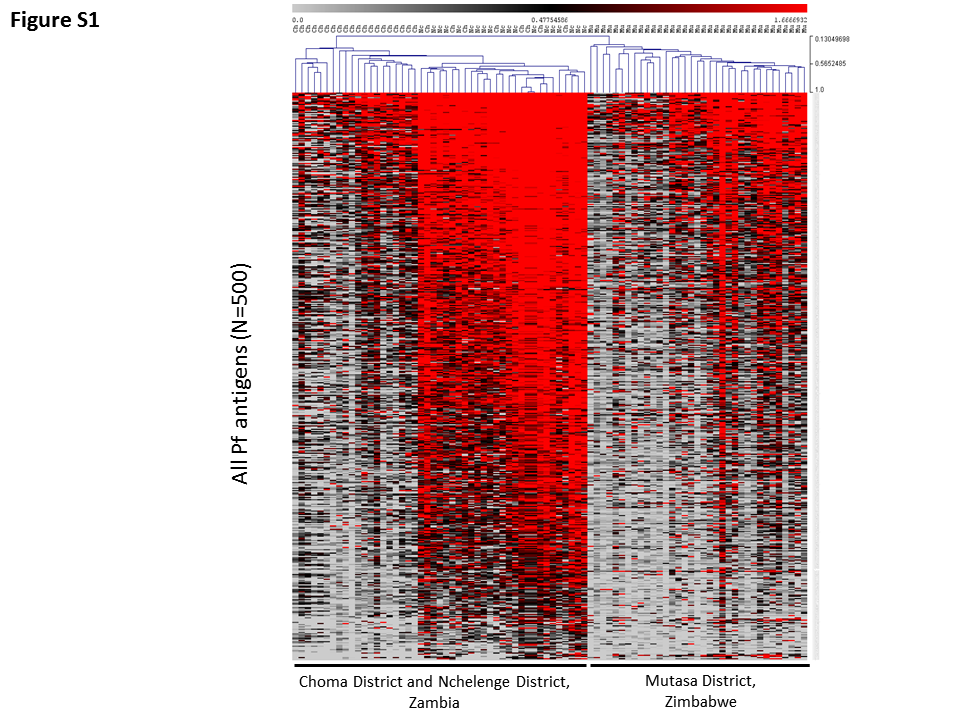

Supplement: FIG S1 [file mSphereDirect.00061-19-sf001.tif]

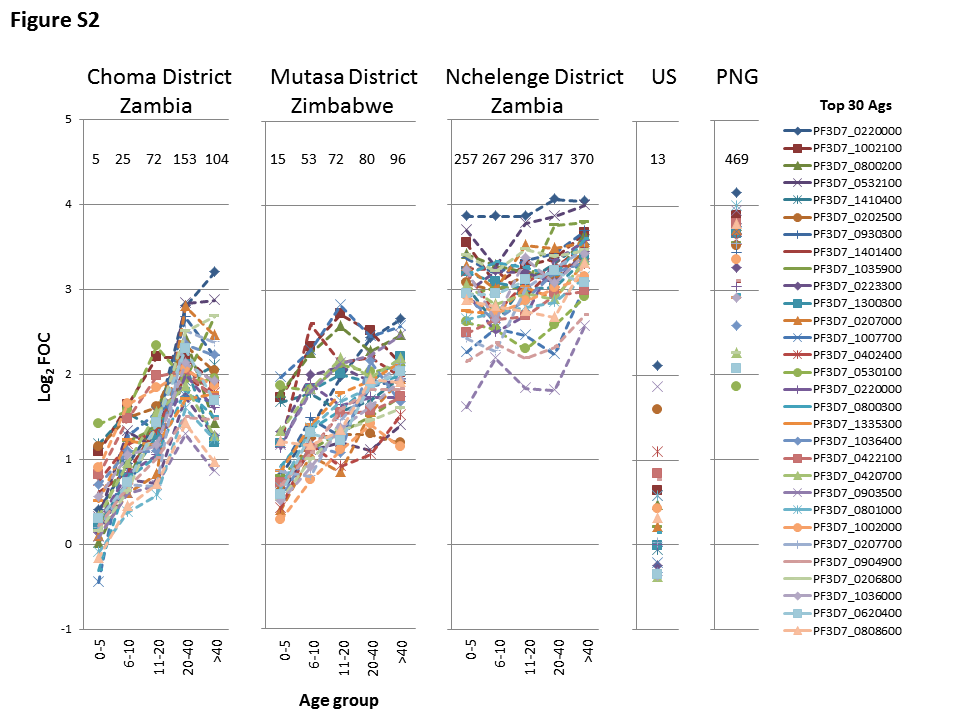

Supplement: FIG S2 [file mSphereDirect.00061-19-sf002.tif]

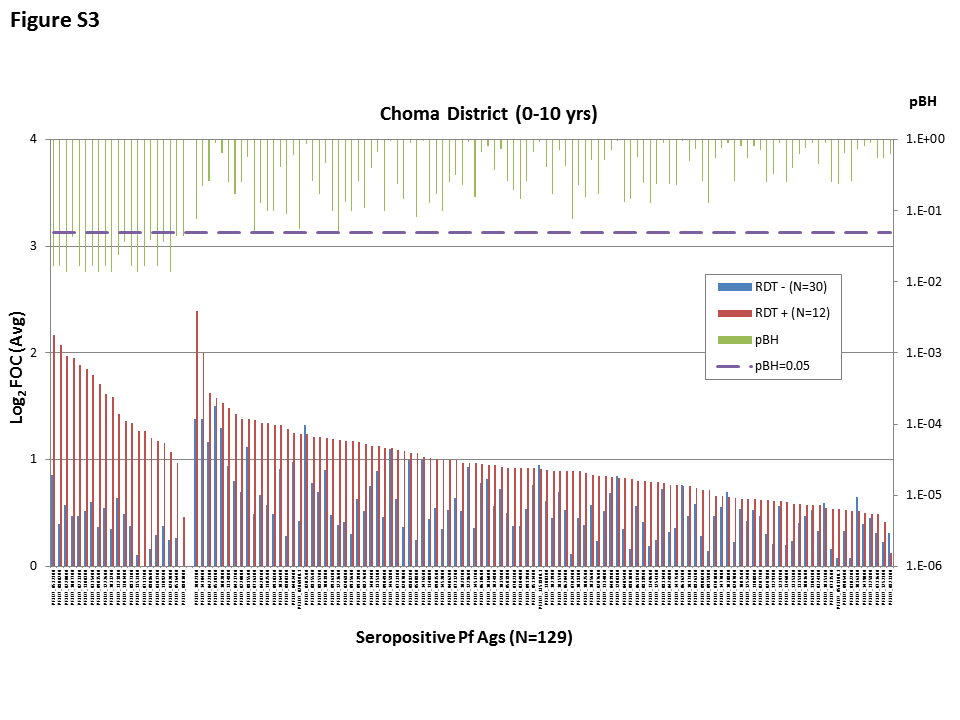

Supplement: FIG S3 [file mSphereDirect.00061-19-sf003.tif]

**Table S2**


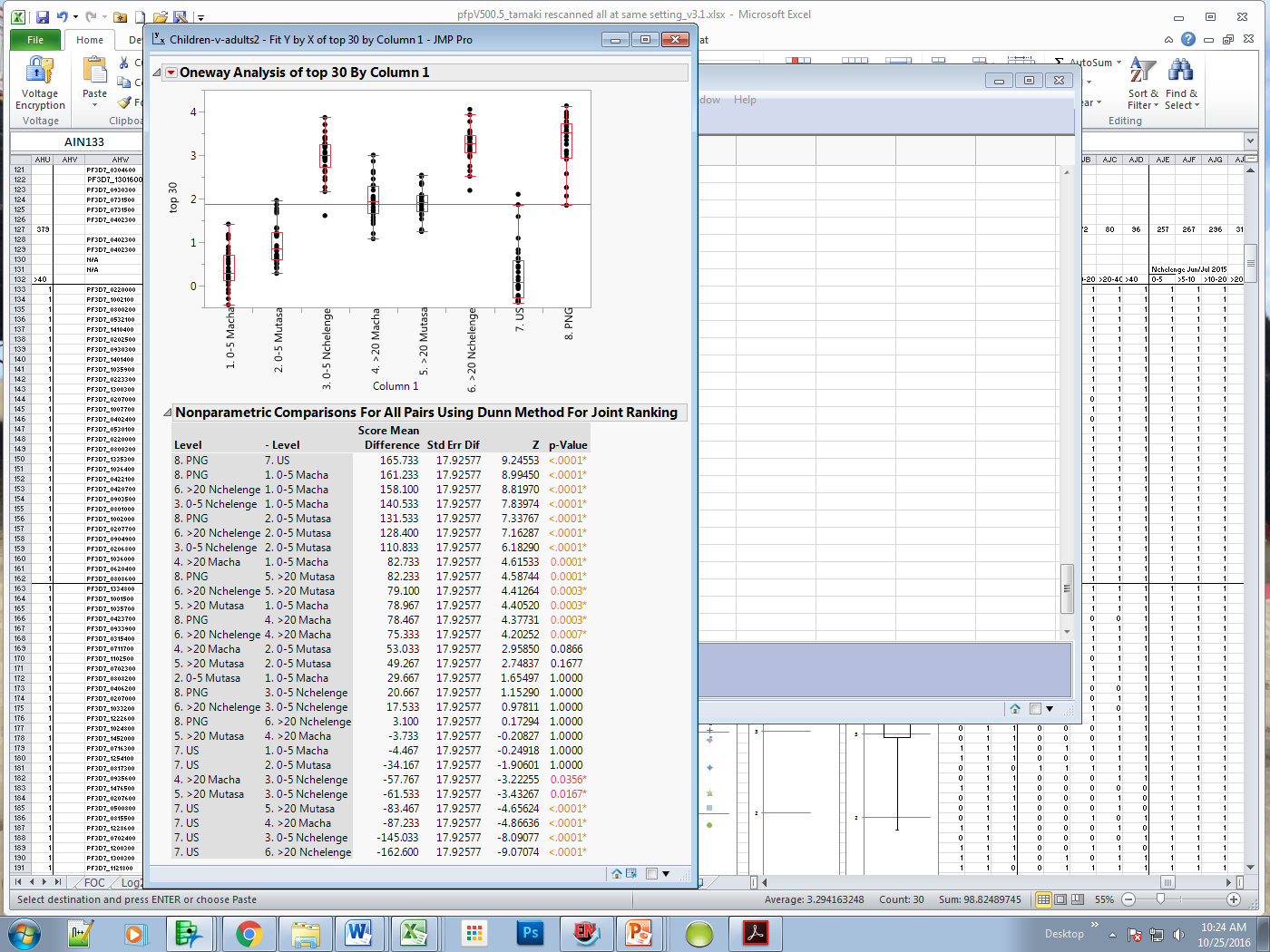

Supplement: TABLE S2 [file mSphereDirect.00061-19-st002.docx]

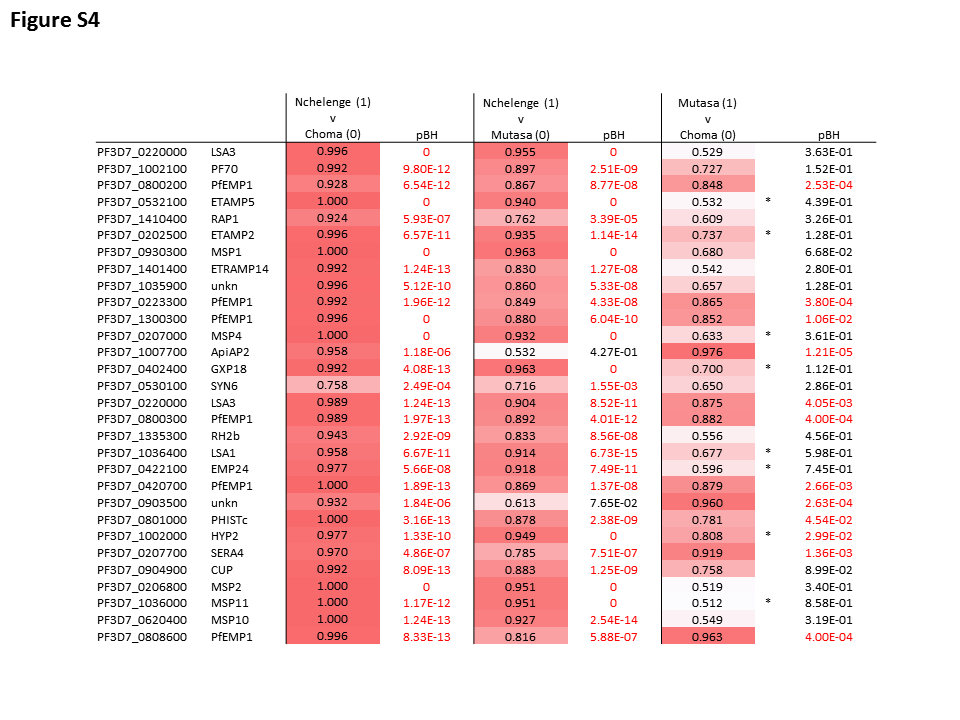

Supplement: FIG S4 [file mSphereDirect.00061-19-sf004.tif]

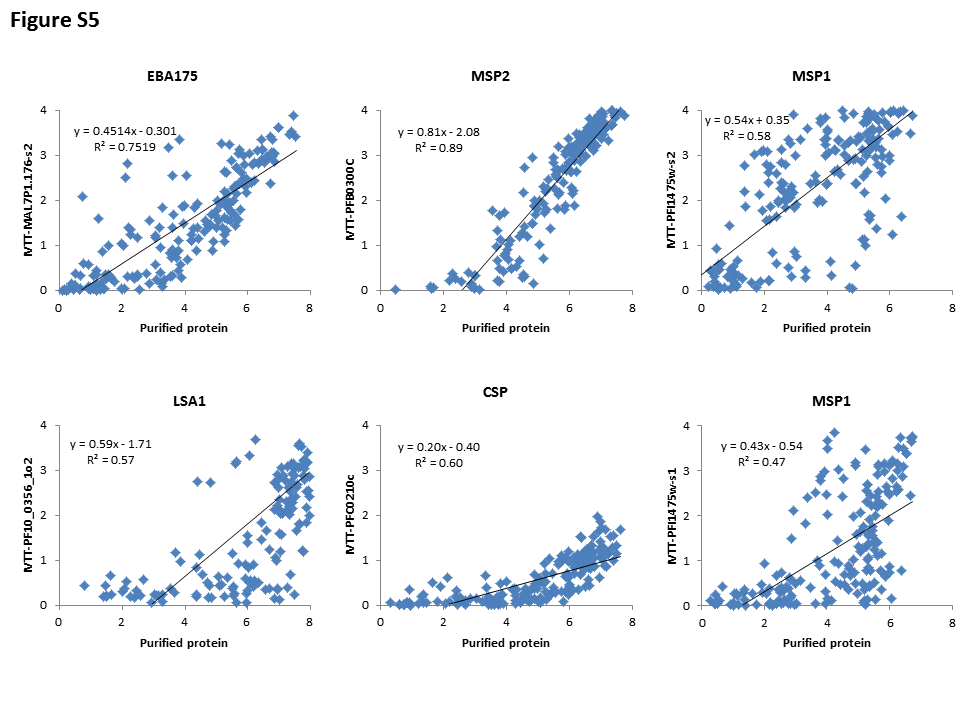

Supplement: FIG S5 [file mSphereDirect.00061-19-sf005.tif]

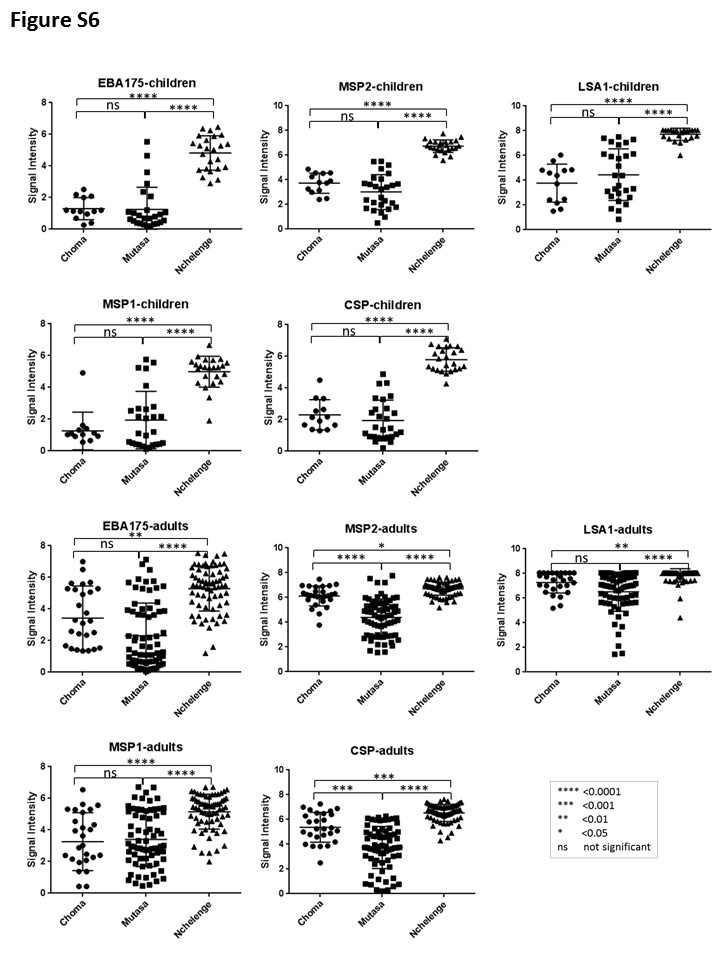

Supplement: FIG S6 [file mSphereDirect.00061-19-sf006.tif]
