# Supplementary material for: Distinct Antibody Signatures Associated with Different Malaria Transmission Intensities in Zambia and Zimbabwe
Source: mSphere. 2019 Mar 27;4(2):e00061-19. doi: 10.1128/mSphereDirect.00061-19 (PMC6437277; doi:10.1128/mSphereDirect.00061-19)
Supplement: TABLE S1 [file mSphereDirect.00061-19-st001.docx]

**Table S1**

| **Gene ID** | **Exon product** | **Name** | **Rank**  **FOC** | **Rank**  **MM** |
| --- | --- | --- | --- | --- |
| PF3D7_0220000 | Exon 2 Segment 1 | Liver stage antigen 3 | 1 | 1 |
| PF3D7_1002100* | Exon 2 of 2 | EMP1-trafficking protein | 2 | 2 |
| PF3D7_0800200 | Exon 2 Segment 1 | Erythrocyte membrane protein 1, PfEMP1 | 3 | 3 |
| PF3D7_0532100 | NA | Early transcribed membrane protein 5 | 4 | 4 |
| PF3D7_1410400* | Exon 1 Segment 1 | Rhoptry-associated protein 1 | 5 | 6 |
| PF3D7_0202500 | Exon 1 of 1 | Early transcribed membrane protein 2 | 6 | 26 |
| PF3D7_0930300 | Segment 2 | Merozoite surface protein 1 | 7 | 9 |
| PF3D7_1401400 | Exon 1 of 1 | Early transcribed membrane protein 14.1 | 8 | 10 |
| PF3D7_1035900 | Exon 1 of 1 | Probable protein, unknown function | 9 | 5 |
| PF3D7_0223300 | Exon 2 | Erythrocyte membrane protein 1, PfEMP1 | 10 | 11 |
| PF3D7_1300300 | Segment 2 | Erythrocyte membrane protein 1, PfEMP1 | 11 | 12 |
| PF3D7_0207000 | Exon 1 of 2 | Merozoite surface protein 4 | 12 | 8 |
| PF3D7_1007700 | Exon 1 Segment 2 | AP2 domain transcription factor AP2-I | 13 | 7 |
| PF3D7_0402400* | Exon 2 of 2 | Plasmodium exported protein, unknown function | 14 | 27 |
| PF3D7_0530100 | Exon 2 of 2 | SNARE protein, putative | 15 | 22 |
| PF3D7_0220000 | Exon 2 Segment 2 | Liver stage antigen 3 | 16 | 13 |
| PF3D7_0800300* | Exon 2 Segment 1 | Erythrocyte membrane protein 1, PfEMP1 | 17 | 14 |
| PF3D7_1335300 | Exon 1 Segment 2 | Reticulocyte binding protein 2 homologue b | 18 | 15 |
| PF3D7_1036400 | Exon 1 of 2 | Liver stage antigen 1 | 19 | 16 |
| PF3D7_0422100 | NA | Transmembrane emp24 domain-containing protein, putative | 20 | 29 |
| PF3D7_0420700 | Exon 2 Segment 1 | Erythrocyte membrane protein 1, PfEMP1 | 21 | 20 |
| PF3D7_0903500 | Exon 1 Segment 1 | Conserved Plasmodium protein, unknown function | 22 | 31 |
| PF3D7_0801000* | Exon 2 Segment 1 | Plasmodium exported protein (PHISTc), unknown function | 23 | 25 |
| PF3D7_1002000* | Exon 2 of 2 | Plasmodium exported protein (hyp2), unknown function | 24 | 33 |
| PF3D7_0207700 | Exon 2 of 4 | Serine repeat antigen 4 | 25 | 17 |
| PF3D7_0904900 | Exon 1 Segment 1 | Copper-transporting ATPase | 26 | 46 |
| PF3D7_0206800 | NA | Merozoite surface protein 2 | 27 | 19 |
| PF3D7_1036000 | Exon 1 of 1 | Merozoite surface protein 11 | 28 | 18 |
| PF3D7_0620400 | Exon 1 of 1 | Merozoite surface protein 10 | 29 | 21 |
| PF3D7_0808600 | Exon 2 Segment 1 | Erythrocyte membrane protein 1, PfEMP1 | 30 | 28 |
| PF3D7_1035700 | Exon 1 Segment 1 | Duffy binding-like merozoite surface protein |  | 23 |
| PF3D7_0933900 | Exon 4 of 4 | Conserved Plasmodium protein, unknown function |  | 24 |
| PF3D7_1001500 | Exon 1 of 1 | Early transcribed membrane protein 10.1 |  | 30 |
