# Supplementary material for: Distinct Antibody Signatures Associated with Different Malaria Transmission Intensities in Zambia and Zimbabwe
Source: mSphere. 2019 Mar 27;4(2):e00061-19. doi: 10.1128/mSphereDirect.00061-19 (PMC6437277; doi:10.1128/mSphereDirect.00061-19)
Supplement: TABLE S3 [file mSphereDirect.00061-19-st003.docx]

**Table S3**

| **ORF** | **Gene ID** | **PlasmoDB**  **PF3D7_** | **Name** | **Size (aa)** | **Length:**  **IVTT** | **Length:**  **Purified Protein ^a^** | **R^2^** |
| --- | --- | --- | --- | --- | --- | --- | --- |
| PFB0300C | PFB0300C | 0206800 | MSP2 | 273 | Full-length | Full-length | 0.89 |
| MAL7P1.176-s2 | MAL7P1.176 | 0731500 | EBA175 | 1,502 | Segment 2 (C-terminal 719aa of exon1) | Conserved domain (Regions III-V) | 0.75 |
| PFC0210c | PFC0210c | 0304600 | CSP | 398 | Full-length | Full-length | 0.6 |
| PFI1475w-s2 | PFI1475w | 0930300 | MSP1 | 1,720 | Segment 2 (C-terminal 868 aa) | MSP1-19-GST | 0.58 |
| PF10_0356_1o2 | PF10_0356 | 1036400 | LSA1 | 1,162 | Segment 1 of 2 (N terminal 250aa) | Internal repeat truncated | 0.57 |
| PFI1475w-s1 | PFI1475w | 0930300 | MSP1 | 1,720 | Segment 1 (N terminal 870aa) | MSP1-19-GST | 0.47 |

^a^ EBA175, MSP1, MSP2, and MSP3 expressed in *E. coli* (James Beeson, Burnet Institute, Melbourne, Australia) (31); CSP expressed in *E. coli* by Gennova Biopharmaceuticals, Ltd (Ashley Birkett, PATH MVI) (28, 29); LSA1 expressed in *E. coli* (David Lanar, WRAIR) (30).
